# Disrupted Ipsilateral Network Connectivity in Temporal Lobe Epilepsy

**DOI:** 10.1371/journal.pone.0140859

**Published:** 2015-10-21

**Authors:** Lorena Vega-Zelaya, Jesús Pastor, Rafael G. de Sola, Guillermo J. Ortega

**Affiliations:** 1 Clinical Neurophysiology, Hospital Universitario la Princesa, Madrid, Spain; 2 Neurosurgery, Hospital Universitario la Princesa, Madrid, Spain; 3 Instituto de Investigación Sanitaria Hospital Universitario de la Princesa, Madrid, Spain; Hospital for Sick Children, CANADA

## Abstract

**Objective:**

The current practice under which patients with refractory epilepsy are surgically treated is based mainly on the identification of specific cortical areas, mainly the epileptogenic zone, which is believed to be responsible for generation of seizures. A better understanding of the whole epileptic network and its components and properties is required before more effective and less invasive therapies can be developed. The aim of the present study was to partially characterize the evolution of the functional network during the preictal-ictal transition in partial seizures in patients with temporal lobe epilepsy (TLE).

**Methods:**

Scalp and foramen ovale (FOE) recordings from twenty-two TLE patients were analyzed under the complex network perspective. The density of links, average path length, average clustering coefficient, and modularity were calculated during the preictal and the ictal stages. Both linear–Pearson correlation–and non-linear–phase synchronization–measures were used as proxies of functional connectivity between the electrode locations areas. The transition from one stage to the other was evaluated in the whole network and in the mesial sub-networks. The results were compared with a voltage-dependent measure, namely, the spectral entropy.

**Results:**

Changes in the global functional network during the transition from the preictal to the ictal stage show, in the linear case, that in sixteen cases (72.7%) the density of the links increased during the seizure, with a decrease in the average path length in fifteen cases (68.1%). There was also a preictal and ictal imbalance in functional connectivity during both stages (77.2% to 86.3%). The SE dropped during the seizure in 95.4% of the cases, but did not show any tendency towards lateralization. When using the nonlinear measure of functional connectivity, the phase synchronization, similar results were obtained.

**Conclusions:**

In TLE patients, the transition to the ictal stage is accompanied by increasing global synchronization and a more ordered spectral content of the signals, indicated by lower spectral entropy. The interictal connectivity imbalance (lower ipsilateral connectivity) is sustained during the seizure, irrespective of any appreciable imbalance in the spectral entropy of the mesial recordings.

## Introduction

Temporal lobe epilepsy (TLE) is the most common type of epilepsy. Fortunately, it has the best prognosis [1] in drug-resistant patients for whom surgery is the only curative/palliative alternative. Careful and precise evaluation of each individual patient is mandatory when defining the minimum cortical zone to be resected during surgery in order to eliminate or reduce the frequency of disabling seizures [2]. This zone, called the epileptogenic zone (EZ), is operationally defined because there is no diagnostic modality able to determine its exact size and location. If the patient is seizure-free after surgery, then the EZ was included in the resected cortex.

This zone-oriented approach, a very successful methodology employed in the current surgical treatment of epilepsy, has been complemented in recent years [3–6] with a broader perspective that takes into account the whole cortical network, instead. For drug-resistant TLE, one can translate the zone-oriented concept of the EZ into the framework of the network approach as a key property -albeit still unknown- of the limbic system to produce/sustain seizures. Whether the network can be altered during the surgery in such a way that its capability to produce/sustain seizures is destroyed, the key property has been destroyed. This is the reason why much effort has recently been devoted to analyzing and characterizing the epileptic network underlying neurophysiological recordings, in TLE and in extra-temporal seizures [6–11] (see also [12] and references therein).

One of the key topics in any complex network is the issue of how connectivity is organized. In particular, functional connectivity -inferred from neurophysiological recordings- is the basic structure on which the accessed cortical network should be constructed. Previous studies have addressed the issue of connectivity distribution in TLE using either interictal neurophysiological recordings [13] or resting-state functional magnetic resonance imaging (fMRI) [14–15]. Although these studies are based on very different techniques and therefore, analyze different kinds of signals (electrical and blood oxygen level–dependent), in both cases interictal decreased connectivity is found on the ipsilateral side.

In this paper, we analyze changes in the functional network underlying the activity recorded by scalp and foramen ovale electrodes (FOE) in twenty-two drug-resistant TLE patients during the preictal and ictal stages. We show that global (scalp + FOE) network properties follow stereotyped changes during the seizure. In particular, the density of lines (DoL) and the modularity (Mod) are the measures with greater changes during seizures. As for the mesial subnetworks, we show the existence of an imbalance between the three most basic network measures during seizures, namely, the DoL, the average path length (APL), and the average clustering coefficient (ACC). The existence of this imbalance was previously reported only during the inter-ictal stage. In the present case we show decreased connectivity in the ipsilateral side during the interictal and also during the ictal stages, producing therefore an altered or disrupted balance of the overall network connectivity. We also show that the imbalance between network measures occurs with no overall mesial changes in spectral entropy (SE). The use of a combined approach of scalp and invasive electrodes (FOE) allows characterizing seizure dynamics in the whole network during partial seizures in TLE.

## Materials and Methods

### Neurophysiological data

The study sample comprised twenty-two patients with TLE (14 women) (Table 1). Mean age was 38.0 years, and the mean duration of intractable epilepsy was 25.8 years. The patients were evaluated before surgery according to La Princesa’s protocol, as previously published [16,17]. This research was approved by the Ethics Committee of the Hospital de la Princesa. Signed written informed consent was obtained from all patients. During the video-EEG recordings, antiepileptic drugs were progressively removed from the second day to the fourth day (approximately one-third of the dose per day). In Table 1, we present clinical information, the results of the pre-surgical studies and the overall physician’s diagnosis regarding lateralization. When the surgery was performed, the type of surgery and outcome (Engel scale) [18] were also included. Patients not requiring resective surgery were categorized as Engel scale 0. One typical seizure per patient was analyzed, that is, twenty-two seizures were included in the present study.

**Table 1:** 

Patients/seizure					Pre-surgical studies			Diagnosis and Surgery		
Patient	Sex	Age (years)	History (years)	Freq.	SPECT	MRI	v-EEG (inter/ictal)	Diag	Cx	Out come
A	Fe	30	16	w	R AM	R MS	BiM(R>L)/RM	R	R AMTR	I
B	Fe	21	9	w	Bi AM (R>L)	R MS	RM/RM	R	R AMTR	I
C	Fe	44	6	w	Bi AM (R>L)	M asim (L<R)	RM/RM	R	R AMTR	I
D	Fe	36	35	w	R AM	R MS	RM/RM	R	R AMTR	I
E	Ma	37	6	d	L AM	Normal	RM/RM	R	R AMTR	I
F	Ma	47	26	w	Bi AM (R>L)	R MS	RM/RM	R	R AMTR	I
G	Ma	34	34	m	Bi AM	Bi MS	RM/RM	R	R AMTR	I
H	Ma	48	43	m	L AM	R MS	RM/RM	R	R AMTR	I
I	Fe	34	33	m	Bi AM (L>R)	R MS	RM/RM	R	R AMTR	I
J	Fe	42	28	w	L AM	M asim	BiM(L>R)/LM	L	L AMTR	I
K	Fe	39	30	w	L AM	L MS	BiM(L>R)/LM	L	L AMTR	I
L	Ma	45	24	m	R AM	M asim (R<L)	BiM(L>R)/LM	L	L AMTR	I
M	Ma	54	44	w	Bi AM	Bi MS	biM(L>R)/LM	L	L AMTR	I
N	Fe	22	5	w	L lat	Bi MS	LM/LM	L	L Lat Cort	I
O	Fe	28	27	w	L AM	L MS	LM/LM	L	L AMTR	I
P	Ma	41	40	m	L Fr & AM	L MS	LM/LM	L	-	0
Q	Ma	56	54	d	L AM	L MS	Mult/Mult	Mult	-	0
R	Fe	34	20	w	L Fr & AM	M asim	LM/LM	L	-	0
S	Fe	34	26	irreg	L AM	L MS	LM/LM	L	L AMTR	II
T	Fe	32	17	w	-	Normal	LM/LM	L	L AMTR	II
U	Fe	38	25	w	R AM	Normal	Mult(L Fr)/LM	L	L AMTR	II
V	Fe	41	19	w	L AM	L MS	Mult/LM	L	L AMTR	III

Diag: Diagnosis; Freq: Seizure frequency; Cx: Surgery; daily; w: weekly; m: monthly; R: Right; L: Left; M: Mesial; T: temporal; Fr: Frontal Bi: Bilateral; MS: Mesial sclerosis; Fr: Frontal; lat: lateral; Cort: Cortectomy; Ma: Male; Fe: Female: AM: Antero-Mesial; AMTR: Antero-Mesial Temporal Resection: asim: Asymmetry; Mult: Multifocal

Six-contact platinum FOEs with 1-cm center-to-center spacing (AD-Tech, Racine, USA) were inserted bilaterally under general anesthesia [19,20]. Correct implantation was assured using fluoroscopic imaging in the operating room. We designated FOE1 as the most rostral electrode and FOE6 as the most occipital one. The left FOEs are designated LFOE1 to LFOE6 and the right FOEs are designated RFOE1 to RFOE6. Scalp electrodes were located accordingly with the international 10–20 system.

### Signal and voltage-dependent analysis

Digital EEG and FOE data were acquired at 500 Hz, exported to ASCII format at 200 Hz and filtered at 0.5–60 Hz (for both scalp and FOE recordings). Epochs suitable for numerical analysis lasting approximately thirty minutes were visually selected. Those epochs containing artifacts due to saturated electrical activity, muscle activity, electrode displacements, etc., were discarded. Interictal spikes on the other side were not considered as artifacts and were not eliminated from the recordings, taking into account their little influence on the synchronization calculations, either in the scalp case (less than 1%) or in the FOE case (approximately 10%), as it was previously demonstrated [21]. A sliding non-overlapping window of 1000 data points was moved along the multivariate recording of typically twenty-eight channels (sixteen from scalp and twelve from FOE). Main measures (see below) were calculated in every temporal window. In this way, a temporal analysis of the measures’ dynamic was obtained with a resolution of five seconds (1000 data points at 200 Hz). All derivations (scalp and FOE electrodes) were referenced to (Fz + Cz + Pz)/3. Data were post-processed using the *R* package. Most of the numerical calculations were carried out with our own scripts. Network measures, on the other hand, were calculated using the *R igraph* package [22]. The broadband signal (0.5–60 Hz) was used to estimate functional connectivity between every pair of electrodes time series using both a linear–Pearson- and a non-linear–phase synchronization- measures.

The two used measures to quantify voltage-dependent activity during the preictal and ictal periods were excitability and SE. These measures do not take into account the correlations between different cortical areas. Excitability (defined below) is used mainly for graphical purposes. The SE, on the other hand, is evaluated in the same manner as the network parameters in order to compare signal against network changes.

To quantify irritative and ictal activity, we used the equation of Schindler et al. [23], according to which we calculated the quantity

$$S_{i}(k)=\left|\frac{x_{i}(k+1)-x_{i}(k)}{\Delta t}\right|$$ (1)

for each electrode time series *x*_*i*_, where ∆t = 0.005 (sampling time).

**Fig 1:**
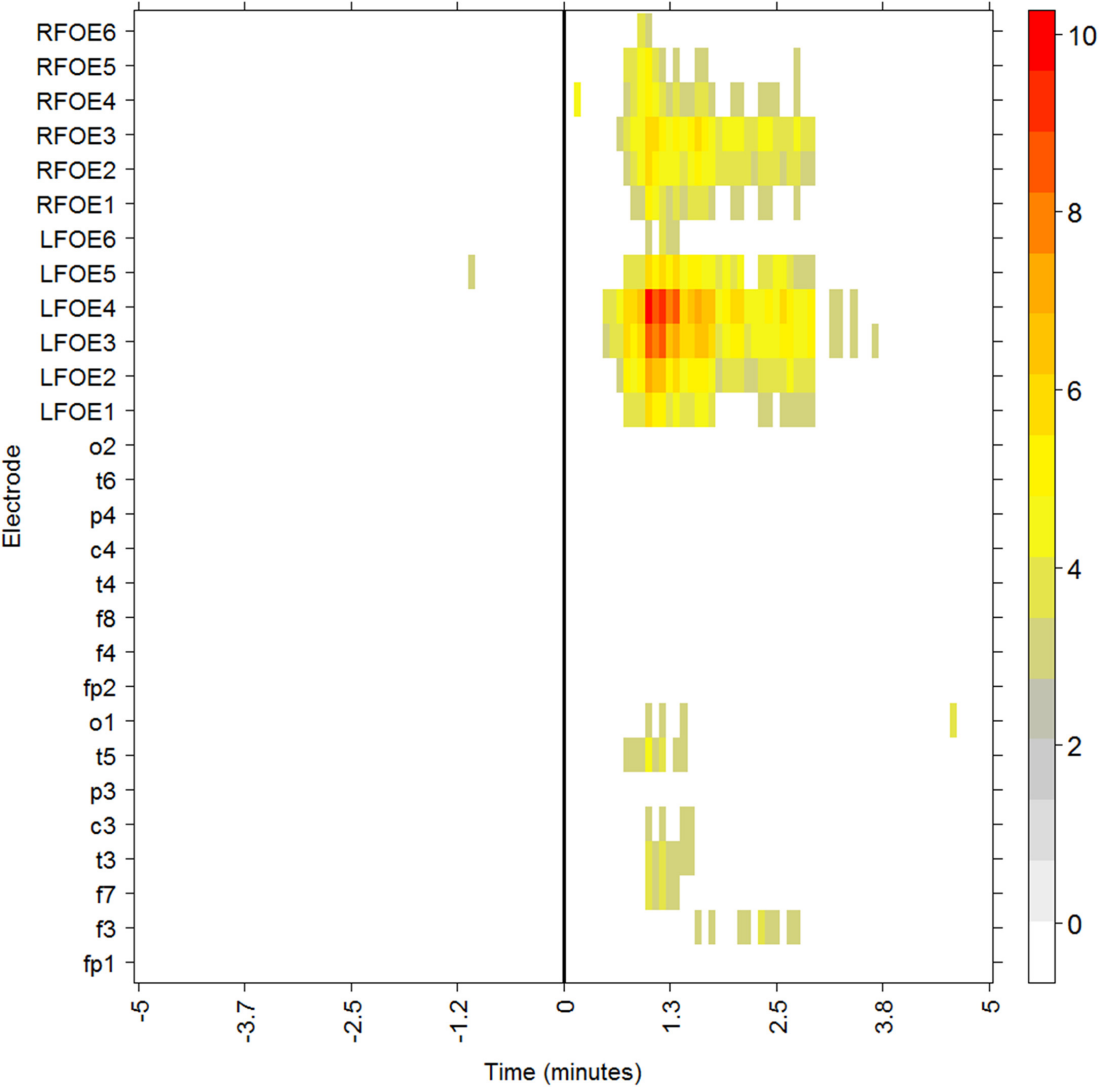
Excitability (Eq 1) for patient J. The color scale quantifies the epileptogenic activity, ${\tilde{S}}_{i}$, for each electrode, *i* (y-axis). The x-axis marks the time relative to seizure onset (as determined by an expert neurophysiologist).

Standard deviations of *S*_*i*_ *(k)* were then calculated for a short baseline period, defined as a period of twelve temporal windows at the beginning of the analyzed epoch, during the preictal stage, as σ_*i*_^*ref*^ *= σ[S*_*i*_ *(k)]* for each channel *i*. *S*_*i*_ *(k)* that was then normalized as ${\tilde{S}}_{i}=\frac{s_{i}(k)}{\sigma^{ref}}$. Consistent with Schindler et al., we found that epileptiform activity was present when ${\tilde{S}}_{i}>2.5$. This threshold value of 2.5 was determined empirically in Schindler et al. and also defined epileptogenic activity well in our study. ${\tilde{S}}_{i}$ is the excitability of channel *i*. A representation of Eq 2 is displayed in Fig 1, which ranges from white (no excitation) to red (high excitation).

The SE, the Shannon entropy of the power spectrum of the signal, was calculated in the following way: For each electrode time series *x*_*i*_, the normalized power spectrum *nPS*_*i*_ is calculated

$$nPS_{i}\left(f_{l}\right)=\frac{PS_{i}{\left(f_{l}\right)}_{}}{\sum_{l}^{}PS_{i}{\left(f_{l}\right)}_{}}$$ (2)

where *PS*_*i*_ *(f*_*l*_ *)* is the power spectrum of *x*_*i*_ and the sum runs all over the frequencies *f*_*l*_. The Shannon entropy of this “probability distribution” is then calculated

$$SE_{i}=-\sum_{l}^{}nPS_{i}{\left(f_{l}\right)}_{}\;\text{log}\;nPS_{i}{\left(f_{l}\right)}_{}$$ (3)

The *SE*_*i*_ so defined is the spectral entropy of channel *i*. The average for either the whole network or the community (see below) was finally calculated as,

$$SE=\frac{1}{N_{chan}}\sum_{i=1}^{}SE_{i}$$ (4)

where *N*_*chan*_ is the number of involved channels. Clinical seizures usually begin with a sudden change in the spectral content of the signals [24], which rapidly dominates the tracing and eventually propagates to other channels. Although the frequencies may be in the alpha range or in the lower or higher bands [25,26], the common characteristic is the existence of a dominant pattern. The SE thus would serves as a good reference of seizure appearance. A shift of the spectral content from multi-frequency–before seizure onset- toward a dominant pattern–during and after the seizure- will lower the SE, regardless of the particular dominant pattern actually presents. In this sense we use the SE as indicator of seizures appearance.

### Network Analysis

In each temporal window of 1000 points (five seconds), cross-correlation was calculated between each pair of time series in all of the twenty-eight electrodes (sixteen EEG electrodes and twelve FOEs). The absolute value of the Pearson correlation coefficient at zero lag between the two time series was calculated as follows:

$$\rho_{ij}=|\frac{\sum_{k=1}^{N_{win}}\left(x_{i}(k)-\bar{x_{i}})(x_{j}(k)-\bar{x_{j}}\right)}{\sqrt{\sum_{k=1}^{N_{win}}{\left(x_{i}(k)-\bar{x_{i}}\right)}^{2}\sum_{k=1}^{N_{win}}{\left(x_{j}(k)-\bar{x_{j}}\right)}^{2}}}|$$ (5)

where $\bar{x_{i}}$ is the mean value of the time series *x*_*i*_ for channel *i* (which has values ranging from one to twenty-eight).

When dealing with neurophysiological data both linear and nonlinear synchronization measures perform similarly [27,28] in many cases, whereas in some cases linear methods probed to be better. Moreover, a detailed study has shown [29] that no synchronization measure performs better than others in models of typical neurophysiological situations. In fact, linear methods, as in Eq 2 perform as good as more sophisticated methods when functional connectivity is estimated. However, when analyzing scalp EEG recordings, as we have done here, volume conduction effects may contaminate the underlying functional connectivity [30,31], especially when it is estimated with a linear and amplitude-dependent method, as it is certainly the Pearson coefficient (Eq 2). In these cases, phase synchronization methods are better suited [31] to deal with this issue. We have thus employed a classical phase synchronization method [32,33] to evaluate connectivity from a nonlinear point of view. The mean phase coherence [33] was calculated in order to obtain values bounded between zero and one which allow us to compare with those obtained through Eq 2. Details about theory of phase synchronization can be found in [32] and its numerical implementation in [34].

A further and most critical step in constructing the functional network relies in assessing whether a link between two different nodes exists or not. Any synchronization measure yields coupling values for any pair of the network nodes, although many of them will not represent actual interactions, that is, they may be not statistically significant. In order to consider only statistically significant links, a common procedure is to establish a particular threshold and then eliminating those links with synchronization values -correlation, phase synchronization, etc.- below that particular value. Numerical procedures to determine links in a more robust and accurate ways have been also published [35]. In this work however, we initially work with a predefined value of threshold equal to 0.5. After that we varied the threshold value in the range 0.1–0.8 in order to scan the behavior of network measures. In this way a clear and complete characterization of the network is performed across a broad range of thresholds [36]. Finally we show that our initial guess of 0.5 was an appropriate value.

The APL and DoL were calculated for the whole twenty-eight nodes network in each epoch. The APL was calculated as the average number of steps along the shortest paths in the network nodes, for all possible pairs of nodes *v*_i_ and *v*_j_, in a network with *n* nodes

$$APL=\frac{1}{n\left(n-1\right)}\sum_{i\neq j}^{}d\left(v_{i},v_{j}\right)$$ (6)

Such that *d(v*_*i*_,*v*_*j*_ *)* is the shortest path between nodes *v*_*i*_ and *v*_*j*_, for every pair of nodes.

The DoL, on the other side, was calculated as the ratio of the actual number of edges in the network to the number of all possible edges between the network nodes.

$$DoL=\frac{\#of\;existing\;links}{\#\;of\;possible\;links}$$ (7)

A network with many links or a high density of links implies highly synchronous behavior at both short and long distances. Although a high DoL decreases the APL in the network because a greater number of edges allows for a greater number of alternative paths between two nodes, the opposite is not always true; that is, a low APL does not always imply a high density of links. A low APL could also be obtained in a network with a few long-distance connections and a large number of local ones. The clustering coefficient characterizes local connectedness in a network by measuring how well neighbors are connected in a given node. Thus, a low value of the average path length could also be obtained in a network with a low density of links, a high average clustering coefficient, and a few long-distance links. This is the well-known small-world property of networks. For a weighted network however, weights should be also taken into account, accordingly with [37]. If *k*_*i*_ is the number of links the node *v*_*i*_ has, the node degree, *s*_*i*_ the sum of the weights of these links, the node strength, and *a*_*ij*_ is either 1 or 0 if a link exists between nodes *v*_*i*_ and *v*_*j*_, the weighted clustering coefficient of node *v*_*i*_ is defined as

$$c_{i}=\frac{1}{s_{i}\left(k_{i}-1\right)}\sum_{j,k}^{}\frac{\left(w_{ij}+w_{ik}\right)}{2}a_{ij}a_{ih}a_{jh}$$ (8)

where *w*_*ij*_ is the value of the “weight” (correlation or phase synchronization values) between nodes *v*_*i*_ and *v*_*j*_.

Therefore, the average of the weighted clustering coefficient ACC is defined for the whole network as

$$ACC=\frac{1}{n}\sum_{i=1}^{n}c_{i}$$ (9)

We also evaluated community structures in the whole network using a well-known community-detection algorithm involving the maximization of modularity [38]. Modularity (Mod) measures how well a given partition or division in a community in a complex network corresponds to a natural or expected sub-division. In our case, we defined the “natural” community structure as the one with four sub-networks corresponding to both mesial areas covered by the FOEs (two communities) and left and right scalp electrodes (two more communities). Thus, the Mod of the network is a measure of how close the actual community structures calculated for each epoch are to the community structure comprising the four regional subnetworks.

Lastly, to evaluate the potential imbalance in subnetworks and SE measures, we calculated the APL, DoL, ACC, and SE for each of the 6-node mesial subnetworks individually.

### Statistical evaluation

To compare the effects of seizure on the network parameters, a paired, one-tailed Wilcoxon signed rank test was used in every case. Size effects were also evaluated by using the standardized mean difference (SMD) as follows

$$SMD=\frac{\text{previous/left measure - after/righ measure}}{\text{pooled standard deviation}}$$ (10)

Previous values–to the seizure onset- in global network measures were compared with the corresponding values after seizure onset. The evaluation was carried out in a temporal window of two minutes, before and after seizure onset. The rationale in this particular choice of the temporal window is explained below (see Results Section). The seizure onset was visually determined by an expert neurophysiologist (LV-Z) in an independent way. Positive values of SMD thus imply decreased values of the evaluated measure. On the other hand, mesial evaluation was carried out by comparing the left-side measure value with the right-side one. In this way, positive values of SMD imply higher measure values in the left side.

## Results

In order to visualize the methodology explained in the Methods section, we first calculated the measures in the neurophysiological recordings from patients J (and patients E, and D in the Supporting Information (S1 File)). We then performed a statistical analysis of the measures behavior of the whole patient sample.

**Fig 2:**
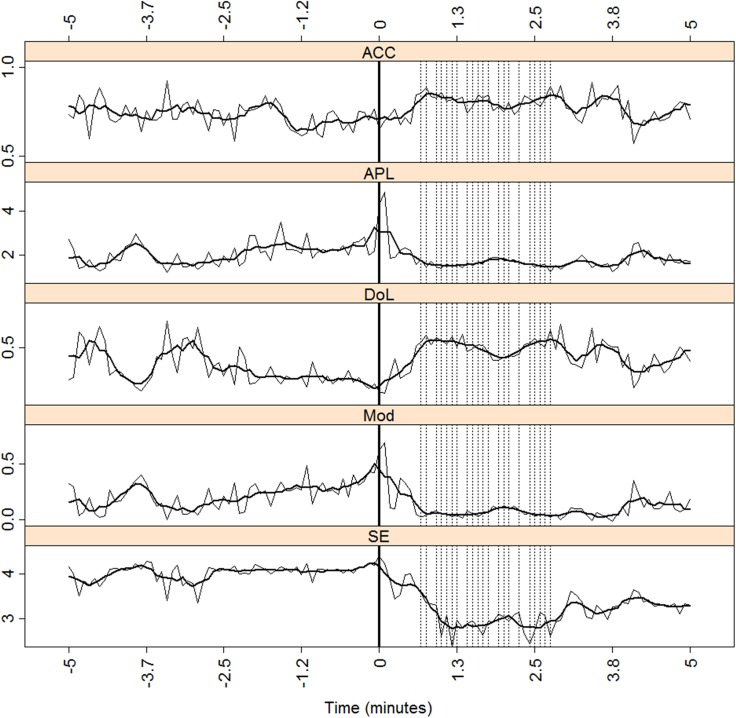
Global measures for patient J. ACC, APL, DoL, Mod and SE for the whole network (scalp + FOE) for patient J. The vertical, dotted lines mark the times when more than four channels reached ${\tilde{S}}_{i}>2.5$ (see text for explanation). The x-axis marks the time relative to seizure onset (thick vertical solid line). A moving average over ten consecutive windows is displayed with a thick solid black line.

Fig 1 shows a typical effect of a seizure in the neurophysiological recordings of patient J (left TLE, see Table 1). Seizure onset, as determined by visual inspection of the raw EEG, is in the origin of times (x-axis). For the whole network (scalp + FOE), we examined the measures ACC, APL, DoL, Mod, and SE during the seizure (Fig 2). In order to better visualize the potential relationship between the excitability and network measures, we included vertical lines (black, dotted) at the times when the excitability was 2.5 times above the background levels (see Methods section) in more than 4 channels. A thick solid vertical line marks seizure onset, as reported by the neurophysiologist (L.V-Z). A moving average is superimposed over ten time steps on the original traces of the measures. Functional connectivity was estimated in this particular case and in the following examples, by using the Pearson correlation (Eq 2) and a threshold of 0.5. A more encompassing study using phase synchronization and different thresholds values is described below.

The ACC (upper panel of Fig 2) increases slightly after seizure onset. The APL decreases immediately after an abrupt and brief elevation during seizure onset. The DoL also increases after onset, although it remains higher than the preictal values, even when the seizure has ended. The ictal values of the other three measures (APL, Mod, and SE) decrease compared with the preictal ones. The fact that the ACC increases and the APL decreases during the ictal period would imply that the functional network structure moves toward a small-network architecture. However, the DoL also increases after seizure onset, such that the changes in the ACC and APL are basically caused by the increasing number of links between the network nodes. The last panel of Fig 2 shows a drop in the value of SE. As can be observed, the SE has an approximately constant value in the preictal period until the seizure appears. At that moment, the SE decreases steadily reaching lower values than during the preictal stage. Moreover, it attains the lowest values after seizure onset with a slowly increasing after that.

**Fig 3:**
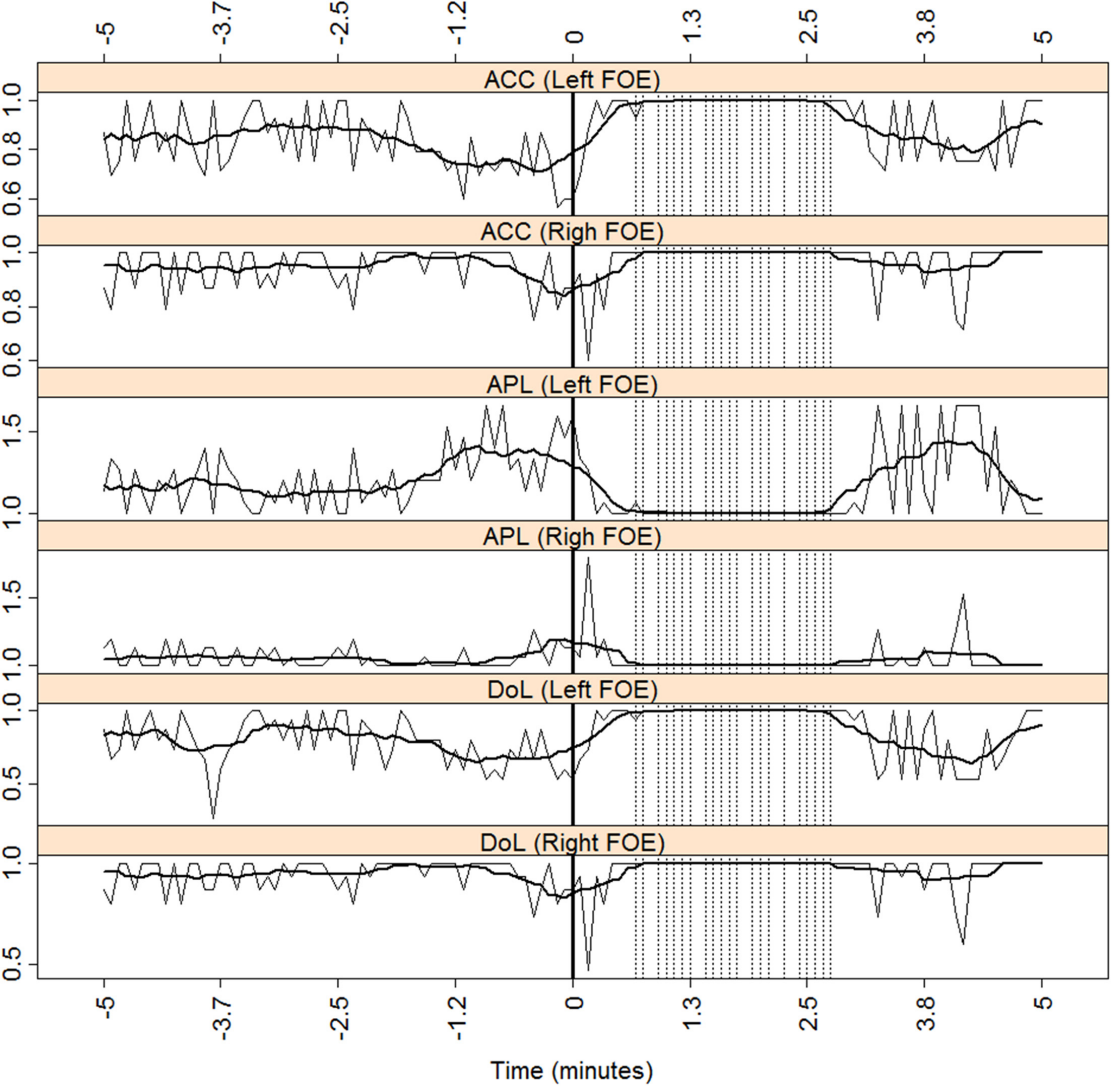
Mesial measures for patient J. ACC, APL and DoL for both the left and the right mesial subnetworks for patient J. The vertical dotted lines mark the times when more than five channels reached ${\tilde{S}}_{i}>2.5$ (see text for explanation). The x-axis marks the time relative to seizure onset (thick vertical solid line). A moving average over ten consecutive windows is displayed with a thick solid black line.

**Fig 4:**
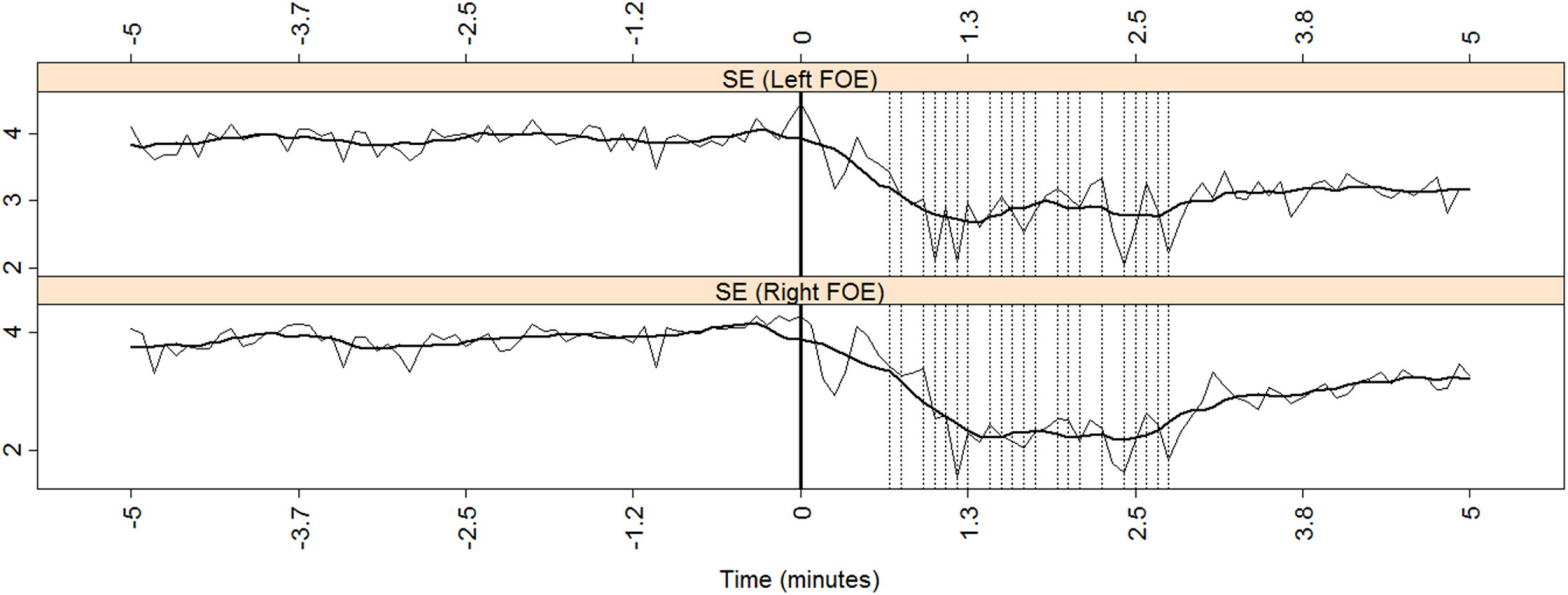
SE for both the left and the right mesial subnetworks for patients J. The x-axis marks the time relative to seizure onset (thick vertical solid line). The vertical dotted lines mark the times when more than five channels reached ${\tilde{S}}_{i}>2.5$ (see text for explanation). A moving average over ten consecutive windows is displayed with a thick solid black line.

To further investigate these findings, we analyzed the effects of the seizure on each temporal side independently, that is, in each mesial subnetwork. Fig 3 displays results that are similar to those in Fig 2, except that this only takes into account the effects of mesial activity, i.e., the effects of cortical activity recorded solely by the FOE on the ACC, APL, and DoL during the preictal and the ictal periods. For APL, the preictal values are higher (on average) on the left side than on the right one. Conversely, the preictal DoL is, on average, lower on the left side than on the right side during the preictal stage. This same observation was recorded with the ACC. Thus, during the preictal period, left ACC < right ACC, left APL > right APL, and left DoL < right DoL. Although it may seem counterintuitive that less synchronization appears on the left side in this left TLE patient (ipsilateral lower DoL and greater APL), the finding is consistent with current knowledge [12,13,14] of decreased ipsilateral interictal synchronization. Lastly, SE did not show imbalances during the preictal or ictal periods, as depicted in Fig 4.

Two other patients, E and D, with right TLE were also analyzed in the Supporting Information (S1 File).

These examples uncover several similarities regarding the network dynamic, both in the global aspects of the whole network, scalp + FOE, and also in the mesial subnetworks. Considering the whole network, the ictal period produces an increment in the values of the ACC and the DoL and a decrease in the values of APL, Mod and SE. These effects are found irrespective of the diagnosis of clinical lateralization (right or left TLE). The behavior patterns of DoL, APL and ACC are interrelated because an increase in the DoL leads to increases (on average) in many neighboring nodes, thus increasing the ACC. The very existence of more links in the network makes the network more “communicable”, thus decreasing the APL. The behavior of these three measures is therefore self-consistent. Modularity adds new information on how the increase in the functional connections is performed. As can be seen in the global measures of the three patients (Fig 2 and A and C in S1 File), Mod and APL behave in a very similar fashion during the preictal period. Furthermore, during the seizures, both measures act in tight correspondence, decreasing after ictal onset and maintaining a very low value throughout the seizure. Because the low value in the APL is a consequence of the increasing number of links between the network nodes, the simultaneous low value in Mod places most of these links between the different modules, or subnetworks, instead of being purely intra-module. In addition, the DoL fails to reach the value one during the preictal or the ictal stages, thus preventing the network from becoming fully connected.

The analysis of the mesial subnetwork measures sheds further light on how connectivity is distributed in these cases. As stated above, the initiation of the ictal period creates more links than those previously existing, and most of them are inter-subnetworks links. However, as can be concluded from Fig 3 (and Figs. B and D in S1 File), there is also an increase in both intra-mesial connections, albeit in a dissimilar way. In the case of patient J (Fig 3), even though a preictal imbalance in the DoL exists (ipsilateral DoL < contralateral DoL), the seizure itself leads both mesial subnetworks to become fully connected networks (ipsilateral DoL = contralateral DoL = 1) almost simultaneously. However, this is not the case for patients E and D (Figs. B and D in S1 File, respectively) because the ictal period does not produce a fully connected network at both mesial sides simultaneously, even though a pre-existent imbalance in the DoL (ipsilateral DoL < contralateral DoL) is found in both patients. It seems that only the contralateral side can reach, and sustain, this condition.

All in all, the above findings naturally lead us to formulate the following hypotheses regarding the whole network connectivity: In TLE patients, seizure onset leads to an increase in the number of inter-regional connections, with a consequent increase in the DoL and ACC and a drop in the values of Mod and the APL. As for the mesial subnetworks, the imbalance in the connectivity distribution leads to lower connectivity on the ipsilateral side than on the contralateral side. This ipsilateral deficit in connectivity occurs both during the interictal period and during the seizure itself.

To test our hypothesis, we evaluated changes in the network parameters using a one-tailed Wilcoxon signed rank test (p < 0.05). The evaluation was carried out in a temporal window of two minutes to the left (before) and two minutes to the right (after) of the seizure onset. The choice in the temporal window is motivated by two factors. First, the average duration of TLE seizures is close to the selected value (106 seconds [39]). And second, many changes in measures begin at seizure onset and last well beyond seizure end (for instance the DoL). In the case of mesial networks we evaluated the imbalance in measures during the preictal period (two minutes prior to seizure onset). An identical procedure was carried out for the ictal case (two-minutes after onset). The potential imbalance in the DoL, APL, Mod and SE was so evaluated.

Therefore, the evaluations were divided in two groups. The first group of comparisons corresponds to the whole network, and the second group to the mesial subnetworks. The (alternative) hypotheses tested in the first group, with respect to the preictal values were:

H_1_: ACC increases during the seizure
H_2_: APL decreases during the seizure
H_3_: DoL increases during the seizure
H_4_: Mod decreases during the seizure
H_5_: SE decreases during the seizure

The proposed hypotheses are not only based on the results found in the analyzed cases but also on previously published results on ictal network dynamics [7,8]. Therefore, it is expected that the ACC and DoL would increase (H_1_ and H_3_) with respect to the preictal values. The increase in the DoL would increase the number of available paths and lower the APL (H_2_). The increase in DoL is expected to make inter-regional links more available and thus lower modularity (H_4_).

**Fig 5:**
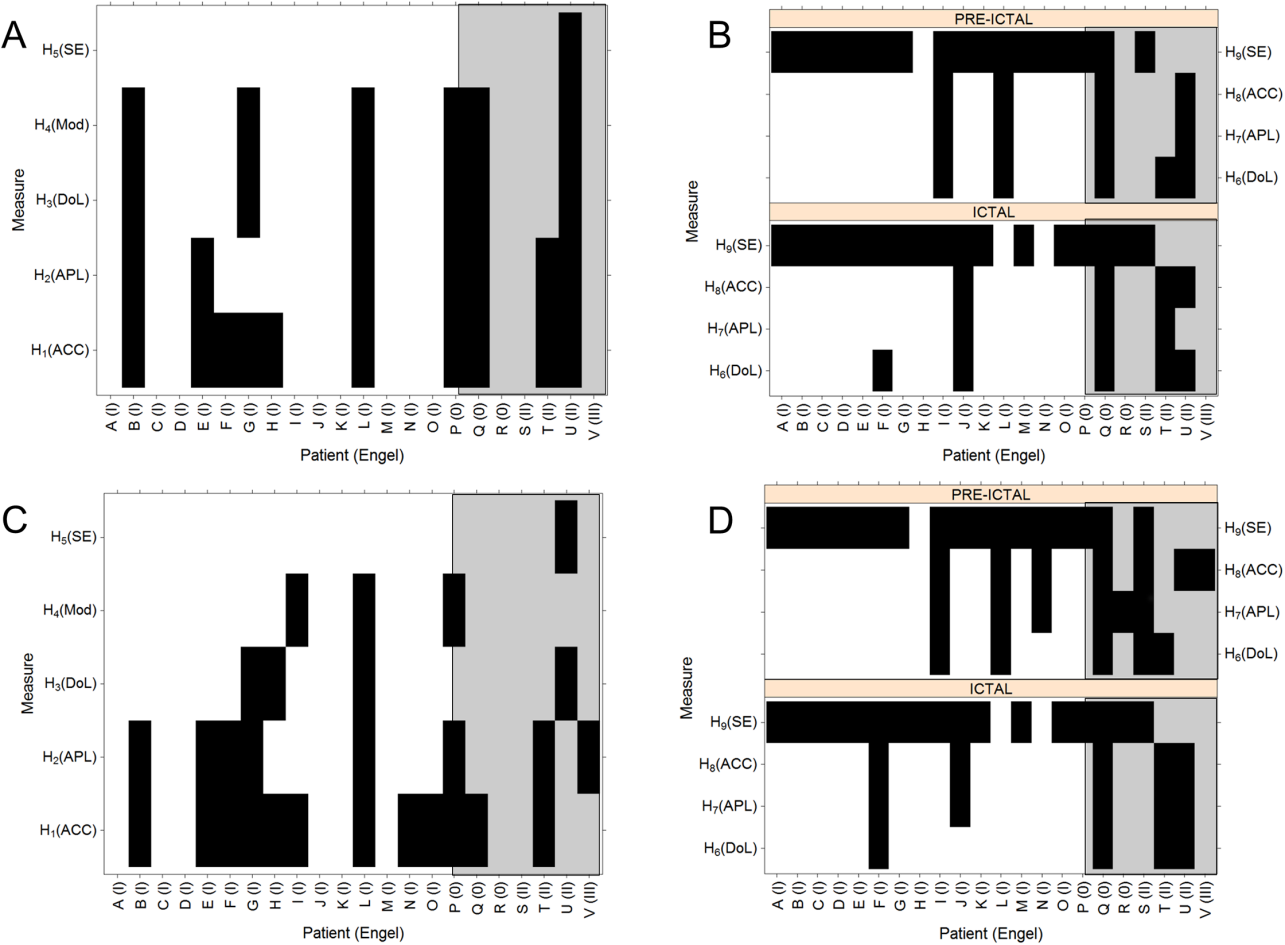
Statistical assessment of global changes and mesial imbalance. Black bars display non-significant (P > 0.05) changes according to the hypothesis used in each case (see text). The x-axis displays the patient number (see Table 1) and Engel classification after surgery. A “0” Engel score means that no resective surgery was performed (patients P, Q and R). (A): Pearson correlation. Global measures (scalp + FOE) during the preictal to ictal transition. (B) Pearson correlation: Mesial measures during the preictal and ictal stages. (C): Phase synchronization. Global measures (scalp + FOE) for the preictal to ictal transition. (B) Phase synchronization: Mesial measures during the preictal and ictal stages.

These results are displayed in Fig 5. Panels A (Global measures) and B (Mesial measures) correspond to network changes when functional connectivity was estimated by using the Pearson correlation and a threshold in the links of 0.5 With a confidence level of 0.05, black regions denote non-significant changes according to hypotheses H_1_, H_2_, H_3_, H_4_ and H_5_. This is to say that changes in measures obeying a particular hypothesis (y-axis) for a particular patient (x-axis) are left blank.

DoL and Mod match the hypotheses in 16 out 22 patients (72.7%), APL in 15 (68.1%), ACC in 12 (54%) and SE in 21 (95.4%).

The best fit to the proposed hypotheses for the network measures corresponds to the DoL, Mod and APL, implying that seizure is characterized by an increase in global synchronization that is not necessarily associated with inner changes in the subnetworks. The fact that ACC does not increase in the same way as APL decreases supports the above conclusion. The decrease in the value of SE is in some way expected because seizures begin with an abrupt change in the signals’ spectral content. This change is usually in the form of a (rapid) transition to a periodic spectral pattern, which is captured by the low value of SE. Whether this behavior in the signals is a consequence of the underlying functional network changes cannot be solely inferred from this analysis.

The next issue we evaluated was the potential imbalance in the mesial subnetwork measures during both the preictal and ictal stages. In those situations we evaluated the following hypotheses:

H_6_: DoL is lower on the ipsilateral side than on the contralateral side.

H_7_: APL is higher on the ipsilateral side than on the contralateral side.

H_8_: ACC is lower on the ipsilateral side than the contralateral side.

H_9_: SE is higher on the left side than the right side.

In this case, the proposed hypotheses were based on previous results, at least for the ACC, APL and DoL. As we mentioned in the Introduction, previous findings demonstrated impaired connectivity on the ipsilateral side in TLE patients [13,14,15]. Thus, we expected a reduction in the DoL on the ipsilateral side (H_6_) with an increase in the ACC (H_8_) and a decrease in the APL (H_7_). Because we had no *a priori* indications of imbalances in SE, we compared the existence of higher values of SE on the left side (H_9_) with the right side in all patients with right and left TLE.

The upper right panel (B) of Fig 5 (Mesial measures) shows the evaluation of these hypotheses. During both the preictal and the ictal stages, network measures prove to behave accordingly with the proposed hypotheses in most cases (from 77.2% to 86.3%). A clear deficit in functional connectivity is observed on the ipsilateral side during the preictal and the ictal stages. Interestingly, the imbalance in the network measures is independent of any appreciable tendency in the SE.

Also, in panels A and B of Fig 5 we included a distinction between “clean” TLE epilepsy patients (patients A to O) and other cases. The fifteen patients belonging to that group were correctly diagnosed and remained seizure-free (two years) after surgery. The other seven cases (gray areas in both panels A and B) did not underwent surgery (patients P, Q and R) for diverse clinical or surgical reasons or continued to experience seizures (Engels II and III) after surgery. In the later cases, palliative surgeries were performed (patients U and V). Panels A and B of Fig 5 thus show that when only Engel I cases (patients A to O) are considered, the tested hypotheses associated with network changes are much better fitted, especially those associated with mesial imbalance.

In order to assess whether any volume conduction contamination affects measures and network properties calculated using the Pearson correlation, we repeated the same procedure but using phase synchronization instead, with the same threshold of 0.5, as before. These results are plotted in panels C and D of Fig 5. A direct comparison between the changes in the global network measures calculated using the Pearson correlation (Fig 5A) and phase synchronization (Fig 5C) shows some differences. The existing differences show a better fit of the phase synchronization methodology in the DoL and Mod with 86% of matching the hypotheses H_3_ and H_4_, in contrast with the 72,6% corresponding to the Pearson case. The other two network measures show an equivalent matching -54% for ACC- or even a poorer one -36% for the APL-. Of course, the SE behaves in the same way in both cases because does not depend of network parameters.

Fig 5D shows the mesial imbalances when the network functional connectivity is calculated using phase correlation. A direct comparison with the corresponding to the Pearson case, panel B of Fig 5, shows also similar results.

**Fig 6:**
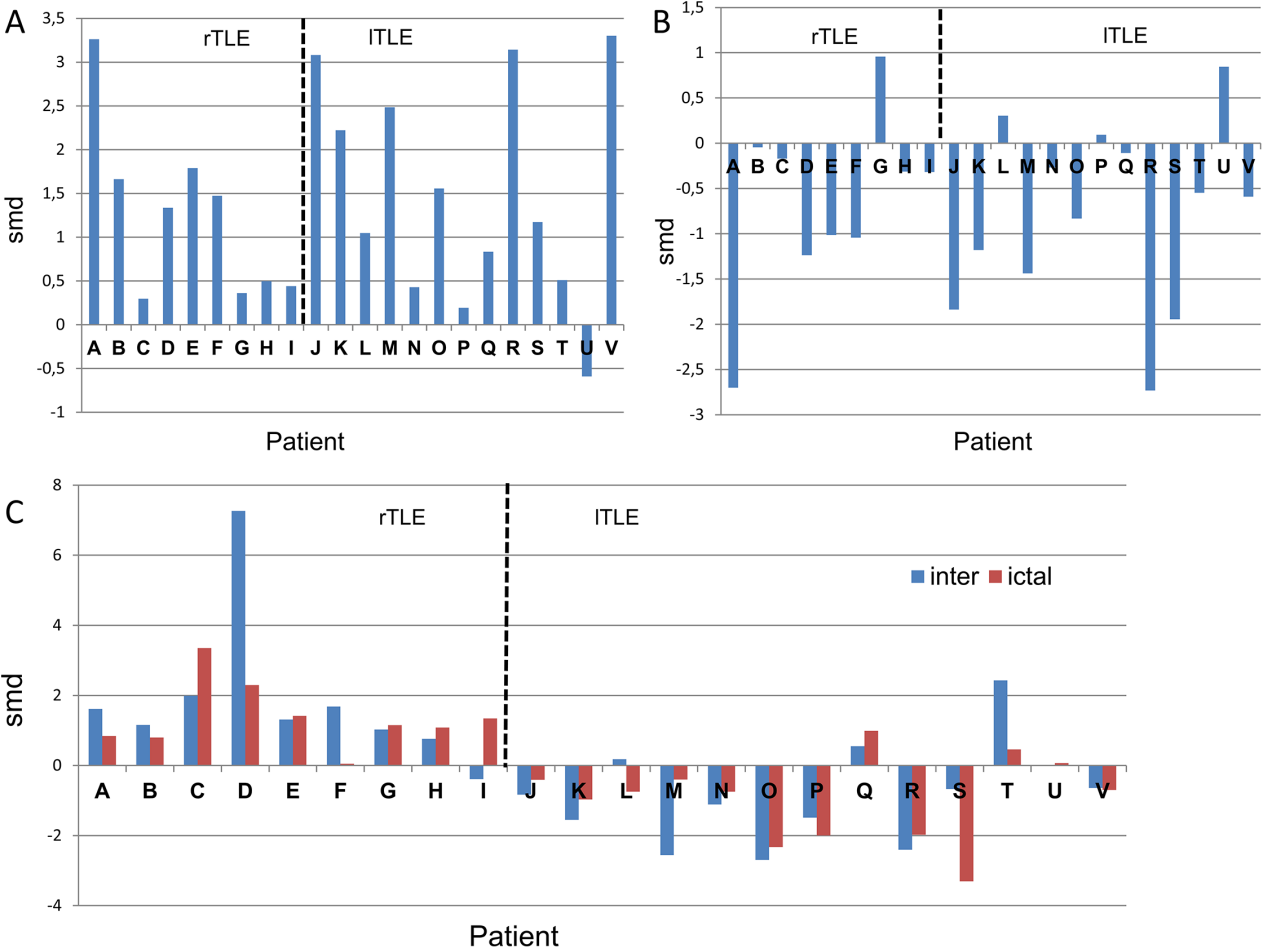
Quantitative comparison of network changes provoked during the seizures. Functional connectivity was estimated using the Pearson correlation. Vertical dashed line separates right from left TLE patients. A: SMD for SE during the preictal-ictal transition. B: SMD for DoL during the preictal-ictal transition. C: Mesial imbalance quantification using SMD for DoL in the interictal (blue) and ictal (red) stages. rTLE stands for right TLE and lTLE stands for left TLE.

A quantitative assessment of the network changes during the preictal-ictal transition was evaluated using the SMD (Eq 10) and depicted in Fig 6. The evaluation was carried out for two global measures; SE (panel A) and DoL (panel B) during the preictal-ictal transition and for mesial imbalance in the DoL during both the preictal and ictal stages (panel C). In this case, the Pearson correlation was used as the functional connectivity measure and a threshold of 0.5 was employed.

**Fig 7:**
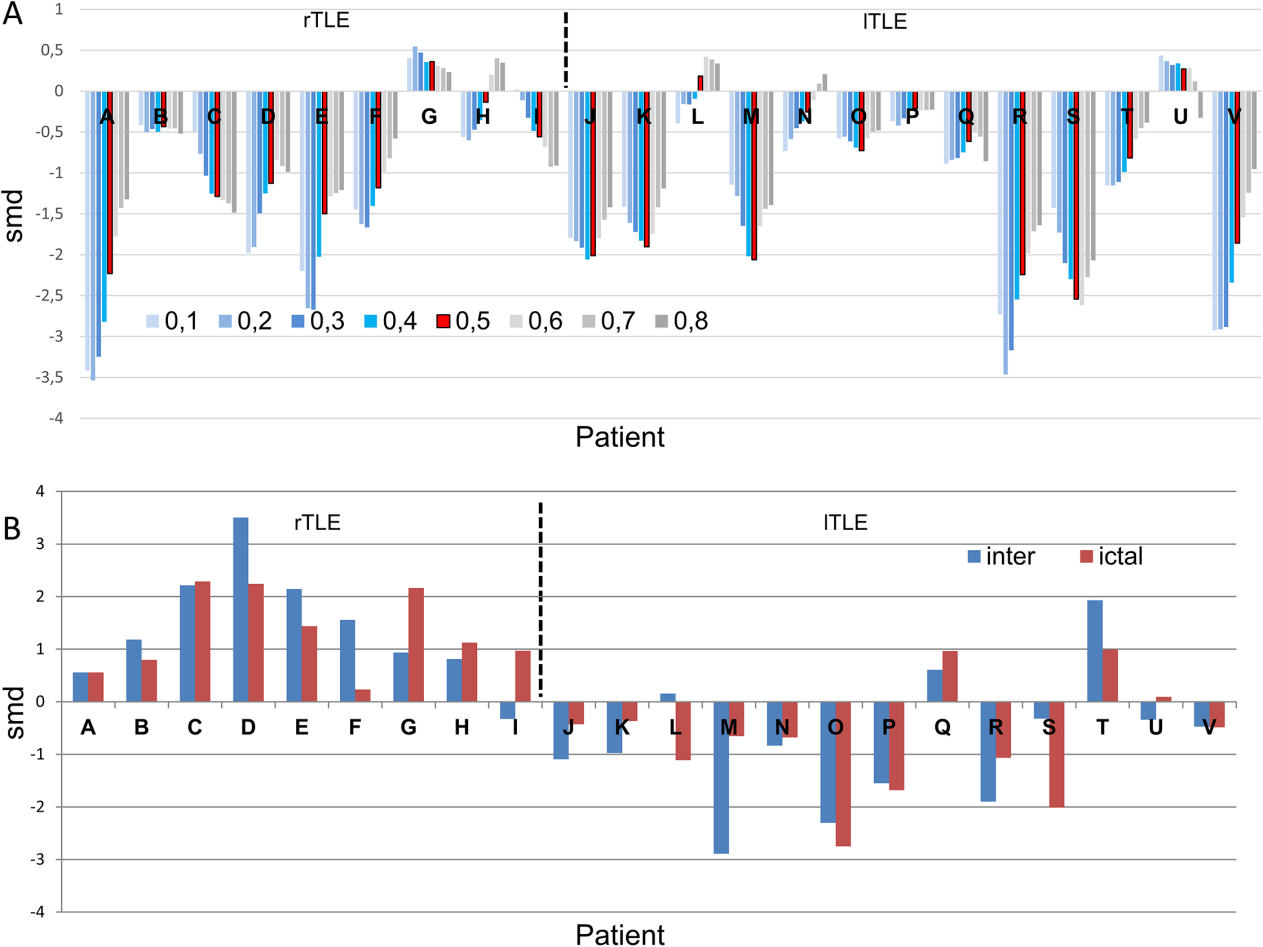
Quantitative comparison of network changes provoked during the seizures. Functional connectivity was estimated by using the phase synchronization. Vertical dashed line separates right from left TLE patients. A: SMD for DoL during the preictal-ictal transition calculated using different values of link thresholds (0.1 to 0.8). B: Mesial imbalance quantification using SMD for DoL in the interictal (blue) and ictal (red) stages. rTLE stands for right TLE and lTLE stands for left TLE.

Until now, all of the network measures were calculated using a threshold of 0.5. As we mentioned in the Methods section, this election is certainly arbitrary and a validation of its used should be accomplished. To do that and following recommendations in [38] we quantify through the use of SMD (Eq 10), changes in the DoL during the preictal-ictal transition, when the network is constructed using the phase synchronization and the thresholds was varied from 0.1 to 0.8. These results are plotted in panel A of Fig 7. For each patient, x-axis, the changes in the DoL in a network constructed using the phase correlation, during the preictal-ictal transition, is evaluated for different values of the threshold. The “arbitrary” value of 0.5 used in the preceding plots is highlighted in red with black borders. A smooth behavior of the SMD values as a function of the thresholds is apparent in almost every case, justifying therefore the preselected value of 0.5. On the other hand, the overall results in this figure should be compared with the equivalent results of panel B in Fig 5, corresponding to the case of Pearson correlation and a threshold of 0.5. Again, results in both figures, panel B in Fig 6 and red strips in panel A of Fig 7, are qualitatively similar.

Lastly, in panel B of Fig 7 we display the mesial network imbalance when phase synchronization is employed as the connectivity measure and a threshold of 0.5, In this sense, this panel should be compared with panel C of Fig 6, which corresponds to the mesial network imbalance when the connectivity is calculated using the Pearson correlation and a threshold of 0.5.

## Discussion

The results of the present study confirm the existence of stereotyped changes in the underlying cortical network during seizures in TLE patients. With respect to the global network (scalp and FOE electrodes), our findings show the existence of increasing global synchronization after seizure onset compared with the preictal stage. This increased synchronization is represented by the increase in the number of links between network nodes and the increase in clustering coefficients. Simultaneously with this rise in global connectivity, the average path length and the modularity decrease. Taking into account that four modules or communities were defined (i.e. left and right FOE and left and right scalp electrodes), a reduction in the modularity value after seizure onset implies that most of the increase in ictal synchronization is due to the appearance of inter-module links. Therefore, it is not surprising that the average path length decreases throughout the network in the ictal phase. The above-mentioned network changes are accompanied by a decrease in the SE, which was used to unify in a single measure the multiple seizure patterns that differed from the early ictal changes (e.g. bursts of spikes and electrodecremental changes), which are commonly observed during TLE seizures [25,40].

Our study also shows that the mesial DoL and the ACC were lower on the ipsilateral side of the seizures than on the contralateral side. This observation was accompanied by an increase in the APL, thus revealing decreased connectivity on the epileptogenic side in this sample of TLE patients. This finding is in accordance with previous studies on interictal recordings [13] and resting state fMRI [14,15]. However, the findings presented here add new information regarding mesial ictal connectivity. As shown, decreased ipsilateral connectivity also persists during the ictal stage. Taking into account that the ipsilateral side encompasses the SOZ in these TLE patients, this fact is also in accordance with similar results recently reported [41] for the cases of extratemporal patients, where the SOZ is the less connected area. However, decreased ipsilateral connectivity is not accompanied by an imbalance (positive or negative) in the SE during the preictal stage or during the seizure. It is worth mentioning that the very existence of interictal epileptogenic activity, which is usually predominant on the ipsilateral side, does not significantly affect synchronization calculations [21]. In any case, whether interictal epileptogenic influence is considered, it would be by increasing ipsilateral synchronization, in contrast to the results presented here.

The comparison between the changes in the network measures, especially the DoL, with the changes in the SE is certainly informative. A decrease in SE in tightly related with seizure appearance (95% of the cases). This fact implies that the spectral content of the signal, on average, underwent a transition from a multi-frequency spectrum before the seizure onset to a narrower content during the seizure. However, this change in the spectral content of the signals is “invisible” to the underlying changes in the mesial sub-networks. As we have shown, no imbalance exists in the SE during the preictal or ictal stages. Therefore, the changes occurring in the spectral content of the signals during the seizures are not associated with the changes in the underlying network.

It is difficult to make a direct comparison with the results of other, related studies that have also analyzed the preictal-ictal transition from a network perspective. First, many of the network papers are based on the analysis of extra and temporal epilepsies with subdural and/or depth electrodes [8,9], which are different from those used in the present study (scalp and FOE in TLE recordings). In some cases, moreover [7], a zoom into the ictal stage is performed, dividing it into three sub-stages, an approach we were unable to adopt. Furthermore, measures and properties other than those analyzed here, for instance *assortativity* [42], are the only ones used. From a network perspective, our findings also differ from results reported elsewhere [6], namely, a shift toward a more regular network (increased ACC and APL) is underwent during the preictal-ictal transition [7,8,9]. Nevertheless, an inner structure is observed in the development of the measures during the seizure itself. In the present study, we found an average increase in the ACC, but not in the APL, after onset. Moreover, the modularity we studied also decreases during the ictal phase, thus supporting the decrease in the APL.

The connectivity imbalance in the mesial areas presented here agree with recent reports suggesting that a loss of connectivity within specific network structures are involved in seizure generation [43,44], not only in TLE but also in the cases of other focal epilepsies [41,45]. Diffusion tensor imaging studies have recently shown [46] a loss of white matter connections in the ipsilateral temporal lobe and limbic system in patients with TLE. A numerical model [44] shows that the likelihood of seizures increases as the network connections are removed, supporting the functional results presented here.

There are, however, specific limitations in the present work. The first one concerns the small number of electrodes recordings, twenty-eight, employed in the construction of the network. This fact is even more severe in the case of the mesial sub-networks with only six electrodes to pinpoint the network dynamic in the cortical area. In this sense thus, the findings presented here should be considered as a coarse-grained approximation of the underlying mesial network, although, as we have shown, capturing essential and critical features. The rather small patient sample, twenty-two subjects, with only one seizure per patient also plays against the robustness of the presented results.

From a methodological point of view it should be remarked the influence of the DoL on the other calculated measures, especially over the APL. Because the use of threshold affects the estimates of the number of links in the network, it will therefore also affect the entire set of network measures. We have tried to minimize this effect by using different thresholds in a wide range of values -0.1 through 0.8- with the objective to characterize the network dynamics in a way as independent as possible of the particular link threshold selected.

An issue which should also be mentioned is in relation to the connectivity analysis performed involving scalp electrodes. Field spread due to volume conduction is always present when scalp electrodes are used [47], and the computation of connectivity/synchronization measures between different electrodes time series should take into account this contaminating effect. One way to overcome this issue has been carried out by several groups [48,49] by attempting to reconstruct a suitable source space from which a functional network is subsequently obtained. The other approach entails the use of measures of synchronization which minimizes the amplitude effects [31], as for instance the Phase Lag Index (PLI). In the present work we followed the second alternative by using the phase synchronization measure, in addition to the Pearson correlation coefficient. As it was demonstrated in [31], the phase synchronization performs at least as well as the PLI in detecting true changes in synchronization between scalp (and FOE), minimizing thus the volume conduction effect.

A recurring issue in the network analysis of epileptic recordings is whether a broadband study should be preferred over the narrow-band signal analysis. Many network analysis present results of connectivity in particular frequency bands and others present similar calculations in the broadband realm, instead. While narrowband analysis is capable of dig deeper into particular aspects of the epilepsy dynamics, it sometimes overlooks important information regarding cross-band interactions. On the other hand, broadband signal analysis could be interpreted as a coarse-grained approximation, overlooking finer details but capturing global aspects of the network connectivity. As reported in a recent meta-analysis [12], many network studies have been performed for the high amplitude-low frequency bands, particularly the theta band, where network alterations in patients with focal epilepsy are most noticeable. In this case, Pearson correlation i.e. the zero-time lag cross-correlation, an amplitude-dependent synchronization measure, seems to be appropriate in order to estimate, at least in a coarse-grained fashion, the functional connectivity between pairs of electrodes time series. Nonetheless, when dealing with highly nonstationary time series, as it is actually the case of recording involving both preictal and ictal dynamics, a finer method should be employed to detect synchronization between time series which contain rapid changes in its spectral content, especially during the seizure stage. In that case, the phase synchronization is best suited to quantify the synchronization between two instantaneous and dominant frequencies even working with broadband signals [50]. The well-matched results between Pearson and phase synchronization estimates of functional connectivity obtained in this work give enough confidence of having captured the essential time-dependent connectivity patterns in each patient network.

From a clinical perspective, the results presented here would reduce dramatically the observational time in the video-EEG room, and therefore will also reduce hospital spending. Usually a TLE patient, candidate for a resective surgery, remains during several days in the video room until enough interictal and ictal (more than one seizure) information is recollected. However, the results presented here show that short periods of interictal recordings would be needed to lateralize the TLE, in most cases. A decreased DoL in one of the two mesial areas, as compared with the other one, would indicate a loss of mesial connectivity in that area. Whether an imbalance with the same sign is obtained during the seizure too, a strong confidence in a correct lateralization should be expected.

The work presented here should be framed into the growing literature on complex network approaches to the understanding of epilepsy, which combined with more traditional methodologies [51] will expect to boost alternative and better treatment for this pathology.

## Supporting Information

S1 FigGlobal measures for patient E.
**ACC, APL, DoL, Mod and SE for the whole network (scalp + FOE) for patient E.** The vertical dotted lines mark the times when more than four channels reached ${\tilde{S}}_{i}>2.5$ (see text for explanation). The x-axis marks the time relative to seizure onset (thick vertical solid line). A moving average over ten consecutive windows is displayed with a thick solid black line.(TIFF)

S2 FigMesial measures for patient E.
**ACC, APL and DoL for both the left and the right mesial subnetworks for patient E.** The vertical dotted lines mark the times when more than five channels reached ${\tilde{S}}_{i}>2.5$ (see text for explanation). The x-axis marks the time relative to seizure onset (thick vertical solid line). A moving average over ten consecutive windows is displayed with a thick solid black line.(TIFF)

S3 Fig Global measures for patient D. **ACC, APL, DoL, Mod and SE for the whole network (scalp + FOE) for patient D**. The x-axis marks the time relative to seizure onset (thick vertical solid line). A moving average over ten consecutive windows is displayed with a thick solid black line.
(TIFF)

S4 Fig Mesial measures for patient D, ACC, APL and DoL measures for both the left and the right mesial subnetworks for patient D. The x-axis marks the time relative to seizure onset (thick vertical solid line). A moving average over ten consecutive windows is displayed with a thick solid black line.
(TIFF)

S1 File Supporting Text. (DOCX)
