# Supplementary figures and images for: Disrupted Ipsilateral Network Connectivity in Temporal Lobe Epilepsy
Source: PLoS One. 2015 Oct 21;10(10):e0140859. doi: 10.1371/journal.pone.0140859 (PMC4619301; doi:10.1371/journal.pone.0140859)

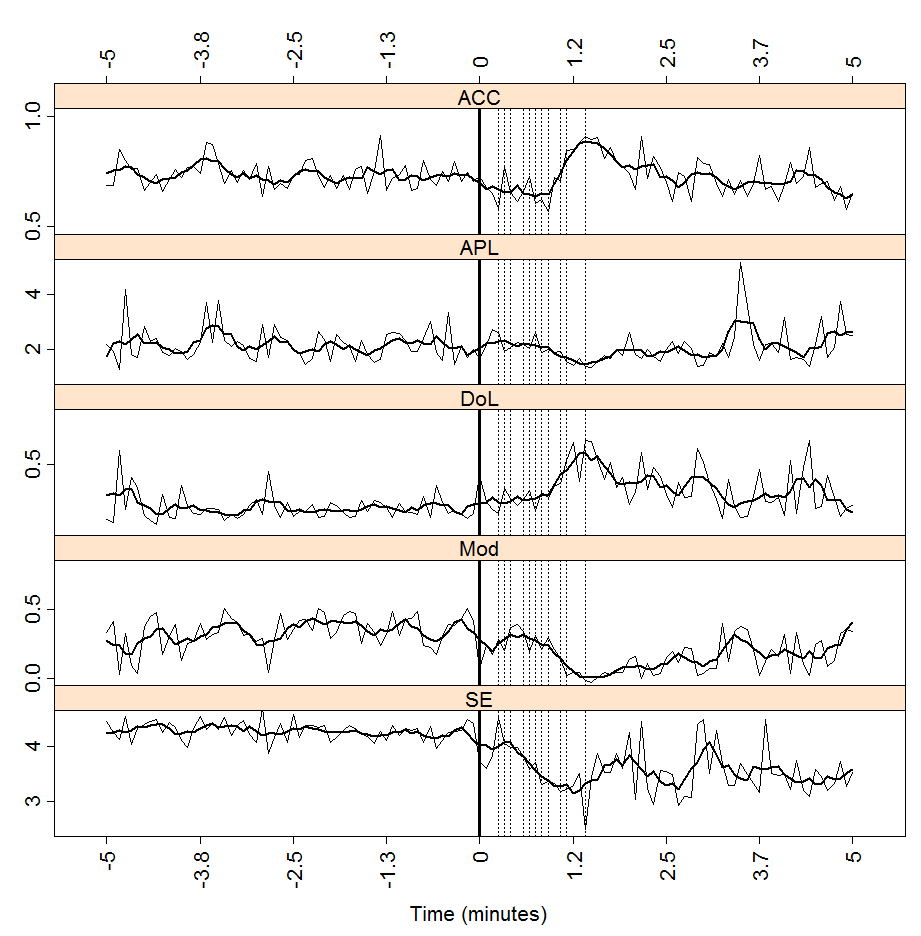

Supplement: S1 Fig — ACC, APL, DoL, Mod and SE for the whole network (scalp + FOE) for patient E. The vertical dotted lines mark the times when more than four channels reached S˜i>2.5 (see text for explanation). The x-axis marks the time relative to seizure onset (thick vertical solid line). A moving average over ten consecutive windows is displayed with a thick solid black line. (TIFF) [file pone.0140859.s001.tiff]

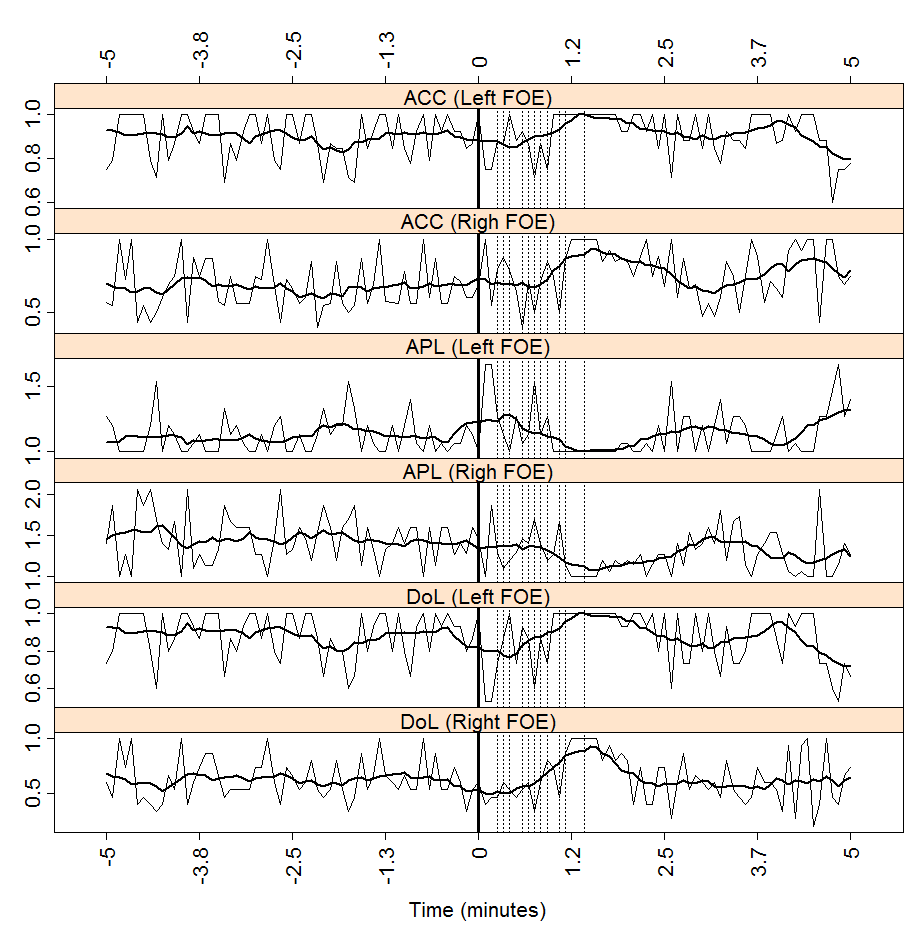

Supplement: S2 Fig — ACC, APL and DoL for both the left and the right mesial subnetworks for patient E. The vertical dotted lines mark the times when more than five channels reached S˜i>2.5 (see text for explanation). The x-axis marks the time relative to seizure onset (thick vertical solid line). A moving average over ten consecutive windows is displayed with a thick solid black line. (TIFF) [file pone.0140859.s002.tiff]

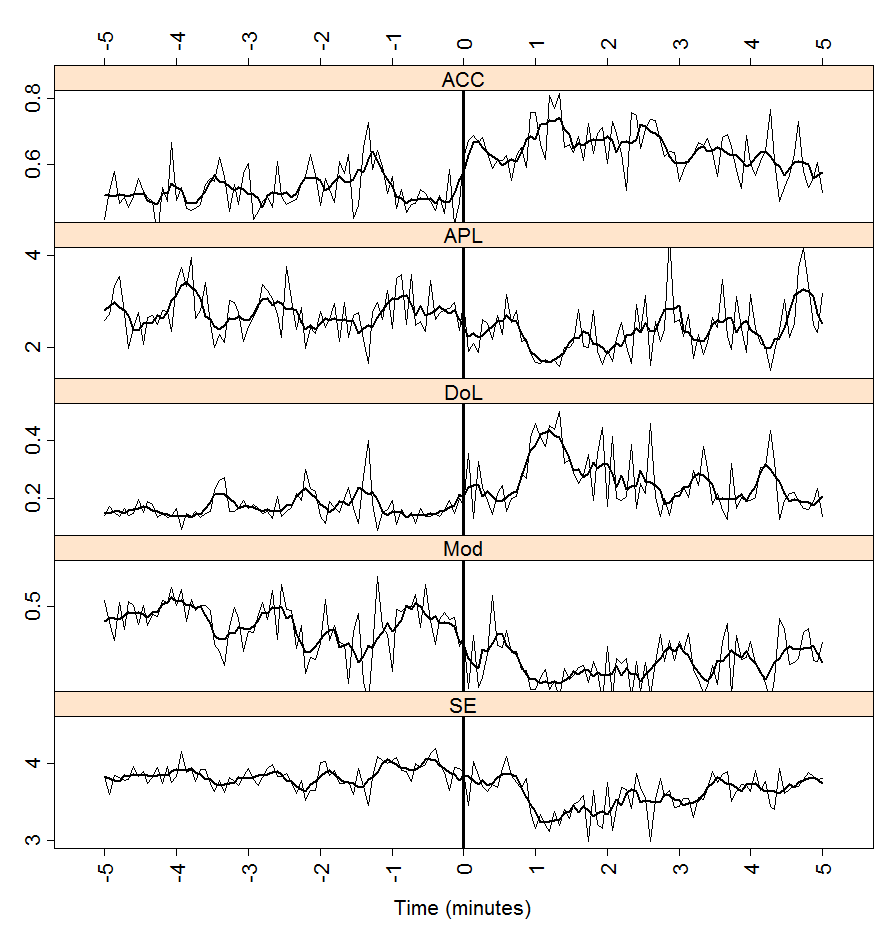

Supplement: S3 Fig — ACC, APL, DoL, Mod and SE for the whole network (scalp + FOE) for patient D. The x-axis marks the time relative to seizure onset (thick vertical solid line). A moving average over ten consecutive windows is displayed with a thick solid black line. (TIFF) [file pone.0140859.s003.tiff]

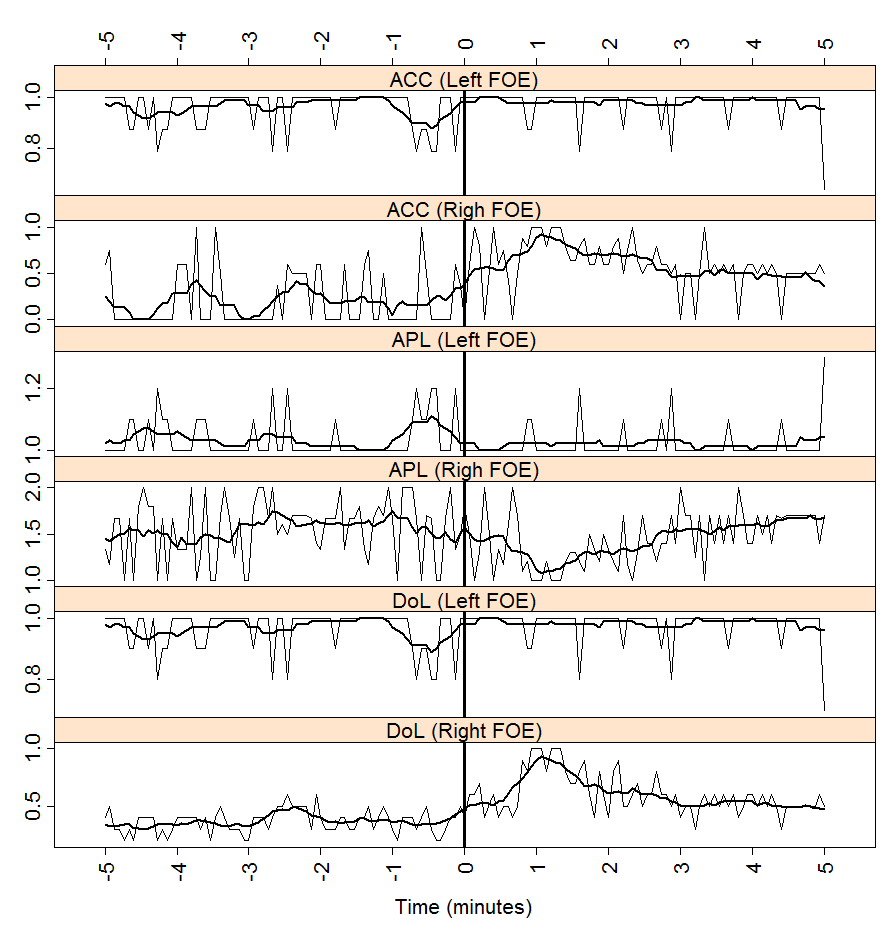

Supplement: S4 Fig — The x-axis marks the time relative to seizure onset (thick vertical solid line). A moving average over ten consecutive windows is displayed with a thick solid black line. (TIFF) [file pone.0140859.s004.tiff]
