# Supplementary material for: Disrupted Ipsilateral Network Connectivity in Temporal Lobe Epilepsy
Source: PLoS One. 2015 Oct 21;10(10):e0140859. doi: 10.1371/journal.pone.0140859 (PMC4619301; doi:10.1371/journal.pone.0140859)
Supplement: S1 File — (DOCX) [file pone.0140859.s005.docx]

## S1 File. Supporting Text

#### Patient E: Excitability, ictal, and interictal imbalance

Patient E (right TLE) has similarities with patient J. Figure A shows the behavior of the global measures during the seizure. The ACC and the DoL increase approximately one minute after seizure onset. The APL, Mod and SE decrease after seizure onset, albeit in different ways. The APL falls slightly compared with Mod and SE, which decrease considerably, with a minimum that coincides with the end of excitability.

Figure B displays the behavior of the mesial measures ACC, APL and DoL. As in patient J, a preictal imbalance can be detected in the three measures, although in the opposite direction to patient J (left ACC > right CC, left APL < right APL and left DoL > right DoL). As explained above, this finding is consistent with lower synchronization on the ipsilateral side in this right TLE patient. Moreover, and in contrast with patient J, an imbalance was also detected in these measures during the seizure. During the period of high excitability, the mesial measures did not attain similar values at both sides. Although the measures undergo changes in the same direction at both temporal sides (ACC increases, APL drops and DoL increases) after seizure onset, extreme values seem to be achieved only on the contralateral side (the left one in this right TLE patient). For instance, this can be seen in the left ACC, which reaches the value one approximately one minute after seizure onset and is sustained longer than on the right side. Lastly, as in patient J, it is noteworthy that extreme values in the network measures are reached once excitability is over.

## Patient D: No excitability, ictal and interictal imbalance

Patient D provides an example of similar behavior in the measures but with no excitability. Figure C displays the global measures during the seizure for patient D (right TLE). The behavior is similar to that of patient E and patient J, with an increase in the ACC and DoL and a decrease in the APL, Mod and SE. In this particular case, however, there is no excitability associated with the seizure, but the patterns in the global measures continue to follow the same rules as before, that is, an increase in ACC and DoL and a decrease in the APL, Mod and SE at seizure onset. Figure D shows the behavior in the mesial areas. As in the two previous cases there is also a preictal imbalance in the three measures (left ACC > right ACC, left APL < right APL and left DoL > right DoL), as in patient E (also a right TLE patient) and in contrast to patient J (a left TLE patient).
